# Sinusite aspergillaire invasive chez un diabétique

**DOI:** 10.11604/pamj.2015.20.279.6596

**Published:** 2015-03-23

**Authors:** Madiha Mahfoudhi, Khaled Khamassi

**Affiliations:** 1 Service de Médecine Interne A, Hôpital Charles Nicolle, Tunis, Tunisie; 2 Service ORL, Hôpital Charles Nicolle, Tunis, Tunisie

**Keywords:** Diabète, sinusite, aspergillus fumigatus, diabetes, sinusitis, aspergillus fumigatus

## Image en medicine

Les sinusites aspergillaires invasives sont des infections fongiques opportunistes rares, rapidement extensives de pronostic redoutable. Elles surviennent préférentiellement sur un terrain d'immunodépression. Leur présentation clinique et radiologique est peu spécifique. Le diagnostic positif est orienté par l'imagerie mais sa confirmation est anatomopathologique et/ou mycologique. Seule une prise en charge médico-chirurgicale rapide et adéquate permettra un meilleur pronostic chez les patients atteints de cette infection. Patient âgé de 60 ans, suivi pour un diabète de type 2, a consulté pour des douleurs rétro-orbitaires gauches avec œdème palpébral homolatéral et fièvre évoluant depuis 7 jours. L'examen physique a trouvé un œdème palpébral, un ptosis et une rougeur oculaire. L'endoscopie nasale a objectivé une muqueuse congestive et nécrosée. L'examen biologique a montré des valeurs glycémiques très élevée. La TDM du massif facial a révélé une sinusite ethmoïdo-maxillaire gauche avec épaississement des tissus mous palpébraux gauches et une infiltration de la graisse extra-conique. Plusieurs diagnostics ont été évoqués notamment un lymphome, une tuberculose ou une néoplasie solide. L'examen mycologique a confirmé la présence de filaments mycéliens d'Aspergillus fumigatus. Il a eu une ethmoïdectomie gauche avec excision des lésions nécrotiques par voie endonasale. Le traitement médical s'est basé sur l'amphotéricine B et l'insulinothérapie pour équilibrer son diabète. L'examen anatomopathologique a conclut à une nécrose de la muqueuse nasale avec présence de nombreux filaments mycéliens. L’évolution était favorable avec un recul de 2 ans.

**Figure 1:**
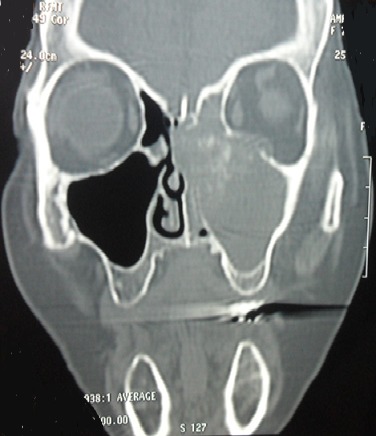
TDM du massif facial: comblement etmoïdo-maxillaire et de la fosse nasale gauche avec lyse osseuse et infiltration de l'orbite

